# Postoperative Bleeding After the Modified Frey Procedure With Laparoscopic Distal Pancreatectomy for a Hemorrhagic Pancreatic Pseudocyst: A Case Report

**DOI:** 10.7759/cureus.82494

**Published:** 2025-04-18

**Authors:** Tadashi Tsukamoto, Tsuyoshi Nishiyama, Chihoko Nobori, Tomohiro Kunimoto, Ryoji Kaizaki

**Affiliations:** 1 Surgery, Osaka City Juso Hospital, Osaka, JPN; 2 Hepato-Biliary-Pancreatic Surgery, Osaka City General Hospital, Osaka, JPN; 3 Surgey, Osaka City Juso Hospital, Osaka, JPN

**Keywords:** frey procedure, hemorrhagic pancreatic pseudocyst, intracystic hemorrhage, pancreatic stone, postoperative bleeding, treatment of chronic pancreatitis

## Abstract

We report a case of a hemorrhagic pancreatic pseudocyst (PPC) in which a laparoscopic distal pancreatectomy and modified Frey procedure were performed. These procedures resulted in postoperative bleeding from a small hole in the pancreatic duct wall, caused by the removal of a pancreatic duct stone.

A 76-year-old man had been undergoing follow-up treatment for six years for alcoholic chronic pancreatitis (CP). While the main pancreatic duct had gradually dilated, and its intraluminal stones had increased in number and size, a PPC had appeared and enlarged gradually at the distal end of the pancreatic tail. During a periodic follow-up, an abdominal computed tomography (CT) scan showed a new, small PPC at the duodenal side of the original, containing a hemorrhagic pseudoaneurysm. Abdominal angiography showed extravasation into the small PPC from arterial branches of the great pancreatic artery, which were subsequently embolized. Nine days after the arterial embolization, a modified Frey procedure with a laparoscopic distal pancreatectomy was performed. The postoperative course was uneventful until 13 days after the operation, when the patient exhibited epigastralgia and melena. An abdominal CT scan revealed hemorrhagic dilatation of the cavity of the longitudinal pancreaticojejunostomy, without pseudoaneurysms or active bleeding. Surgical exploration revealed arterial bleeding from a small hole in the pancreatic duct wall, which had been created during the removal of a pancreatic stone in a previous operation. Hemostasis was achieved through suture closure of the hole, and a pancreaticojejunostomy was performed again. The patient has been alive and well for five years since the surgery, without recurrence of pancreatitis, PPCs, or hemorrhage. The Frey procedure is one of the most common procedures for CP. During this operation, as many stones as possible are removed from the pancreatic duct, which may sometimes be incarcerated in a small branch of the pancreatic duct; therefore, a small hole is sometimes observed after their removal. However, it is unpredictable whether the hole may contact the wall of an arterial branch of the pancreas. Therefore, to prevent postoperative bleeding after the removal of pancreatic duct stones, suture closure of the hole should be considered.

## Introduction

A pancreatic pseudocyst (PPC) is a localized fluid collection containing high concentrations of digestive enzymes in or around the pancreatic tissue, surrounded by a well-defined wall of fibrous granulation tissue, without a true epithelial lining [1]. It is one of the complications of chronic pancreatitis (CP), and is formed after acute exacerbation of this disease or ductal disruption following increased pressure on the pancreatic ductal branch due to obstruction by stenosis, calculi, or protein plugs [2,3]. PPCs often do not require therapy; up to 60% of them resolve spontaneously [4,5]. However, when they are symptomatic or have complications like rupture, intracystic hemorrhage, pseudoaneurysm, or infection, they require therapeutic interventions like endoscopic ultrasound-guided drainage of PPC [6], transcatheter arterial embolization [7], or resection of the PPC [8]. Hemorrhage is reported to occur in 6%-17% of PPCs [7]. Once these occur, they can be lethal. The mortality rate of treated hemorrhagic PPCs is reported to be 13%, while that of untreated hemorrhagic PPCs is reported to be 90% [9].

Here, we present a case of a patient with a hemorrhagic PPC due to CP, treated by laparoscopic distal pancreatectomy and a modified Frey procedure. The Frey procedure is a safe and effective treatment for CP with a dilated duct [10]. It involves combining resection and drainage via a longitudinal pancreaticojejunostomy, with coring-out of the pancreatic head [11]. Laparoscopic distal pancreatectomy is less invasive than the open distal approach [12]. These procedures are generally safe; however, there are some risks associated with postoperative morbidity, including surgical site infection, pancreatic fistula, and postoperative hemorrhage [13]. In this case, postoperative bleeding occurred from the intraluminal surface of the main pancreatic duct.

## Case presentation

**Figure 1:**
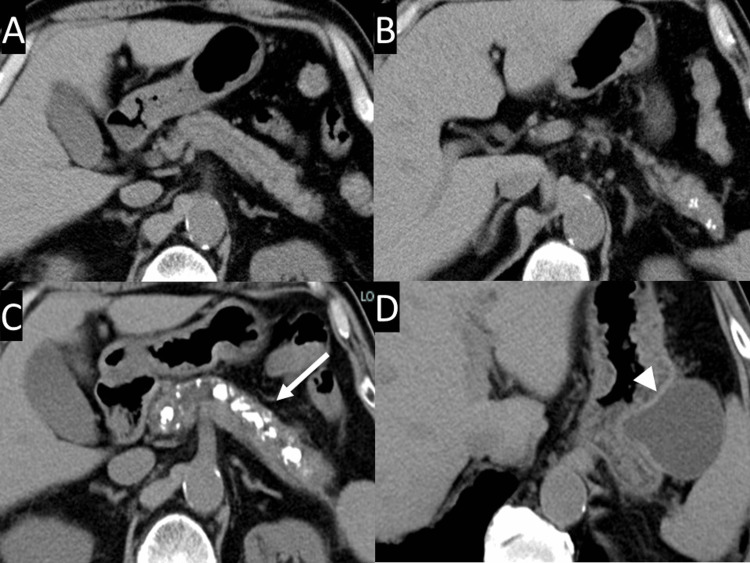
CT scan CT scans 7 years ago (A, B) and 1 year ago (C, D) show that the main pancreatic duct gradually dilates, its intraluminal stones increase in number and size (arrow), and a pancreatic pseudocyst appears and gradually enlarges at the distal end of the pancreatic tail (arrowhead). CT, computed tomography

A 76-year-old man was monitored for six years for alcoholic CP, liver cirrhosis with hepatitis C virus infection, and diabetes mellitus. He had a history of alcohol abuse and smoking. Periodic follow-up abdominal computed tomography (CT) revealed gradual dilatation of the main pancreatic duct, an increase in the number and size of intraluminal stones, and the appearance and gradual enlargement of a PPC at the distal end of the pancreatic tail (Figure 1).

During the follow-up period, the patient displayed no specific symptoms, and the laboratory data from periodic blood tests showed no significant changes (Table 1).

**Table 1:** Clinical course CT, computed tomography

Before and After Admission				Interventions and Events						
7 years before			>	CT revealed some small stones in the pancreatic tail						
											1 year before			>	CT revealed increase of intraluminal stones in the pancreas and enlargement of pancreatic pseudocyst						
Just before			>	CT revealed a new small pancreatic pseudocyst and hemorrhagic pseudoaneurysm within it																	
																						>	The patient exhibited no symptoms						
			On admission			>	Embolization of the hemorrhagic pseudoaneurysm was performed																						
														9 days after			>	Frey operation with distal pancreatectomy was performed						
22 days after			>	The patient suddenly exhibited epigastralgia and melena																				
			>	CT revealed hemorrhagic dilatation of the longitudinal pancreaticojejunostomy cavity						
											>	Surgery for postoperative bleeding was performed						
			29 days after			>	Abdominal angiography showed no aneurysm											

**Figure 2:**
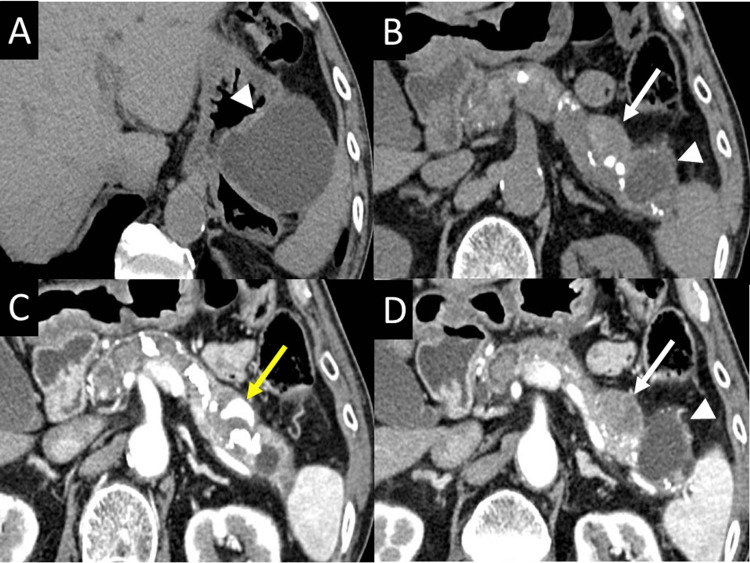
Plain CT scan and arterial phase of dynamic contrast-enhanced CT scan Plain CT scans (A, B) and arterial phase of dynamic contrast-enhanced CT scans (C, D) showing a new, small pancreatic pseudocyst (white arrows) on the duodenal side of the original one (arrowheads), and a hemorrhagic pseudoaneurysm within it (yellow arrow). CT, computed tomography

One year after the last follow-up, CT revealed a new, small PPC measuring 21 mm in diameter on the duodenal side of the original PPC (Figures 2A, 2B, 2D), which contained a hemorrhagic pseudoaneurysm (Figure 2C).

The patient reported no abdominal pain or melena. He was admitted for therapeutic intervention due to the risk of pseudoaneurysm rupture, though his vital signs were stable, and physical examinations revealed no significant findings. Laboratory data were almost within normal range, as illustrated in Table 2.

**Table 2:** Laboratory data on admission and 13 days post-Frey surgery AST, aspartate aminotransferase; ALT, alanine aminotransferase; GGT, gamma glutamyl transferase; CRP, C-reactive protein; WBC, white blood cell count

	Reference Range	On Admission	13 Days Post-Frey Surgery
AST (U/L)	8 - 38	16	14
ALT (U/L)	4 - 44	13	16
GGT (U/L)	16 - 73	17	22
Amylase (U/L)	41 - 112	49	22
Albumin (g/dL)	3.9 - 4.9	3.6	2.3
Blood sugar (mg/dL)	73 - 109	109	110
CRP (mg/dL)	<0.26	0.09	1.49
WBC (/μL)	3170 - 8400	6580	12530
Hemoglobin (g/dL)	11.0 - 14.7	11.8	7.8
Hematocrit (%)	35.2 - 46.7	35.1	22.3
Platelet cell count (10^3/μL)	167 - 390	75	193

**Figure 3:**
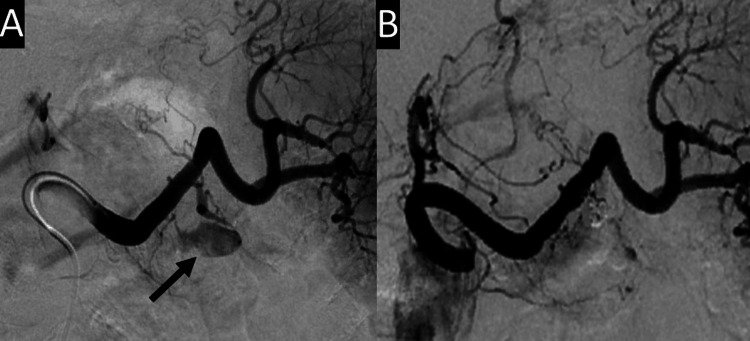
Splenic arteriography Splenic arteriography showing extravasation (arrow) into the small pancreatic pseudocyst from branches of the great pancreatic artery (A), which were embolized (B).

Abdominal angiography on the day of admission revealed extravasation from the pseudoaneurysm of the arterial branch of the great pancreatic artery into the small PPC, which was subsequently embolized (Figure 3).

**Figure 4:**
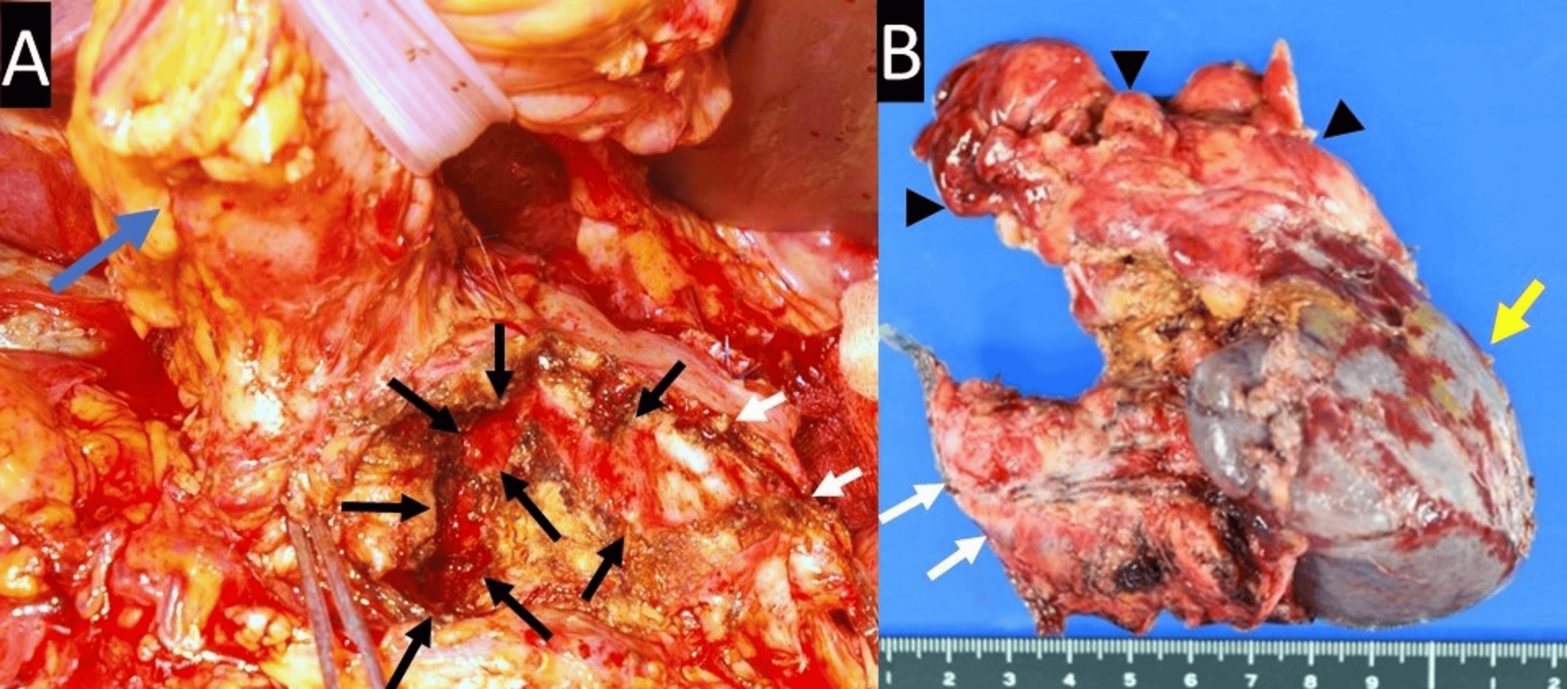
(A) Intraoperative view of the pancreas; (B) Macroscopic view of the resected specimen White arrows depict the cut end of the pancreatic body; Black arrows depict the opened main pancreatic duct; Black arrowheads depict the pancreatic pseudocyst; Blue arrow depicts the stomach; Yellow arrow depicts the spleen.

After embolization, the patient had no abdominal symptoms, and laboratory data showed no significant changes. Nine days after embolization, laparoscopic distal pancreatectomy was performed for hemorrhagic PPC with curative intent, together with a modified Frey procedure on the residual pancreas for CP with stones (Figure 4).

**Figure 5:**
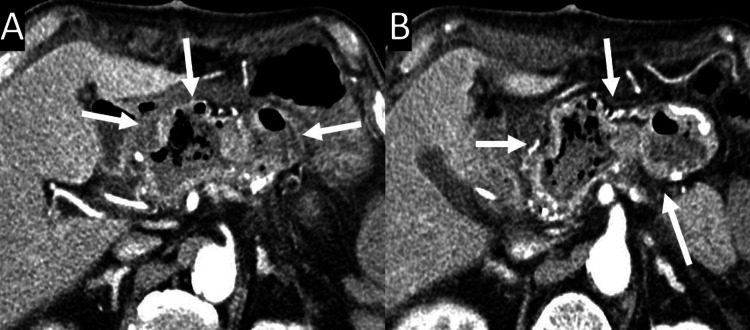
Abdominal CT imaging 13 days after the Frey operation (A)-(B) These images reveal hemorrhagic dilatation of the cavity of the longitudinal pancreaticojejunostomy, without a pseudoaneurysm or active bleeding (arrows). CT, computed tomography

Microscopic examination of the pancreas revealed CP with a PPC and no malignant findings. Hemorrhagic PPC revealed a microscopically dilated pancreatic duct with lacerated ductal epithelium and bleeding, but no aneurysmal findings. The postoperative course was uneventful until 13 days post-surgery, when the patient exhibited epigastralgia and melena. His abdomen was mildly distended, and there was mild tenderness around the epigastrium. The patient’s vital signs were stable. Laboratory data revealed anemia, as illustrated in Table 1. Abdominal CT revealed hemorrhagic dilatation of the longitudinal pancreaticojejunostomy cavity, without pseudoaneurysms or active bleeding (Figure 5).

Gastroduodenoscopy revealed bleeding through the duodenal papillae. Exploratory laparotomy revealed no fluid collection or infection in the abdominal cavity. The jejunum anastomosed to the residual pancreas was dilated, and surgical exploration of the cavity revealed coagula containing a small amount of arterial bleeding from a small hole at the intraluminal surface of the main pancreatic duct, which was caused by the removal of an incarcerated stone in a branch of the pancreatic duct during the previous operation. Hemostasis was achieved by suture closure of the hole, and longitudinal pancreaticojejunostomy was performed again. The postoperative course was uneventful, and there were no morbidities. One week postoperatively, an abdominal angiography revealed normal arterial branches around the pancreas and no aneurysms. The patient has been alive and well for five years postoperatively, without recurrence of pancreatitis, PPCs, or hemorrhages.

## Discussion

The strategy for CP with hemorrhagic PPC is controversial, depending on the location of the PPC, hemorrhagic modalities, and bleeding mechanisms. There are various bleeding modalities for hemorrhagic PPC [7,14-16], such as (i) occurring only in the pseudocyst; (ii) rupture of the hemorrhagic PPC into the peritoneal or pleural cavity; (iii) hemobilia due to penetration of the PPC into the bile duct; (iv) hemosuccus pancreaticus due to the penetration of the PPC into the pancreatic duct; and (v) penetration into surrounding viscera, such as the stomach, small or large intestines, liver, spleen, kidney, or inferior vena cava. The principal etiologies of bleeding are (i) rupture of vessels in the wall of the PPC; (ii) rupture of arteries surrounding the PPC; and (iii) rupture of a pseudoaneurysm into the PPC. Rare etiologies of bleeding include (iv) rupture of a pseudoaneurysm into the pancreatic duct, bile duct, or peritoneal cavity; and (v) rupture of a varicose vein into the digestive system, caused by left portal hypertension due to compression of the splenic vein by the PPC [17]. In our case, the bleeding modality of the hemorrhagic PPC was confined to the pseudocyst, and the etiology of bleeding was likely the threatened rupture of a pseudoaneurysm in the wall of the PPC.

The therapeutic management for hemorrhagic PPC depends on the status of bleeding and circulation dynamics. In cases of unstable hemodynamics due to bleeding, hemostatic treatment with interventional radiology is initially attempted, followed by curative surgery if stable hemodynamic conditions are achieved [14]. Arterial embolization without any additional treatment could be curative; however, 37% of cases that underwent only arterial embolization have been reported to be associated with re-bleeding [14]. In our case, the bleeding status of the hemorrhagic PPC was mild and did not affect the patient’s condition, vital signs, or laboratory data; therefore, angiography and arterial embolization of the pseudoaneurysm could be performed under stable conditions. Thereafter, a laparoscopic distal pancreatectomy could be performed electively with curative intent for the hemorrhagic PPC.

Historically, 40%-75% of patients with CP have required surgery [18]. The greatest indication for surgery in CP is intractable pain. Other indications for surgery are a suspicious neoplasm and local complications of the adjacent organs, such as duodenal or common bile duct stenosis; a pseudoaneurysm or erosion of the large vessels; large PPCs; and internal pancreatic fistulae. The primary goal of surgery for CP is the long-term relief of pain and control of associated complications. However, in some cases, early surgical intervention for CP can preserve the endocrine and exocrine function of the pancreas [19].

Several surgical strategies for CP may be selected according to the clinical conditions or therapeutic goals, which are categorized into three major groups of procedures: decompression of the pancreatic ducts by drainage, resection, and mixed techniques [18]. In this case, a laparoscopic distal pancreatectomy for a hemorrhagic PPC, and a modified Frey procedure with pancreatic duct lithotripsy on the remnant proximal pancreas for CP were both performed.

The Frey procedure, which combines partial resection of the pancreatic head with a lateral pancreaticojejunostomy, is a common surgical procedure. As with other procedures, it is reported to preserve pancreatic endocrine function [10]. A major concern regarding the Frey procedure is the possible development of severe postoperative morbidities. One of them is postoperative hemorrhage associated with a pancreatic fistula or exposure of the cross-sectional aspect of the pancreas to the jejunum [20]. In this case, meticulous hemostasis of the cross-sectional aspect was achieved using cautery in a soft coagulation mode; therefore, postoperative hemorrhage was avoided at the cross-sectional aspect. However, a hemorrhage developed on the intraluminal surface of the main pancreatic duct during the pancreaticojejunostomy. The hemorrhage was suspected to be caused by the exposure of pancreatic juice to the arterial branches in contact with a small pocket, induced by the removal of the pancreatic duct stone. During the Frey procedure, as many stones as possible are usually removed from the pancreatic duct. Stones are sometimes incarcerated in a small branch of the duct; therefore, a small pocket is sometimes observed after the removal of stones. However, whether the pocket may contact the pancreatic arterial branches is unpredictable. Therefore, to prevent postoperative hemorrhage, it is necessary to consider suture closure of the pocket. In this case, liver cirrhosis and diabetes mellitus could be risk factors for postoperative bleeding and might impact the surgical results. A more precise surgical procedure and careful postoperative care should be considered based on the patient’s comorbidities.

## Conclusions

We performed a laparoscopic distal pancreatectomy and modified Frey procedure for CP with a hemorrhagic PPC. Hemorrhagic PPC can sometimes be lethal; however, in this case, arterial embolization of the pseudoaneurysm was performed before its rupture, and the surgery was performed under stable conditions. In the case of CP accompanied by a hemorrhagic pseudocyst, preoperative embolization can facilitate a safer surgical procedure. Postoperative bleeding accompanied the Frey procedure. It was inferred that the pancreatic ductal lithotomy during the Frey procedure exposed the arterial branches attached to the ductal pocket incarcerated by the stone. Therefore, during this procedure, it is necessary to take meticulous care of the pockets on the intraluminal surface of the pancreatic duct, created by the removal of pancreatic duct stones, in order to prevent postoperative hemorrhage. The patient's condition and CT scans should be carefully monitored after surgery to identify any postoperative bleeding early.
